# Mental Health, ART Adherence, and Viral Suppression Among Adolescents and Adults Living with HIV in South Africa: A Cohort Study

**DOI:** 10.1007/s10461-022-03916-x

**Published:** 2023-01-02

**Authors:** Andreas D. Haas, Raphael Lienhard, Christiane Didden, Morna Cornell, Naomi Folb, Tebatso M. G. Boshomane, Luisa Salazar-Vizcaya, Yann Ruffieux, Patience Nyakato, Anja E. Wettstein, Mpho Tlali, Mary-Ann Davies, Per von Groote, Milton Wainberg, Matthias Egger, Gary Maartens, John A. Joska

**Affiliations:** 1 Institute of Social and Preventive Medicine, University of Bern, Mittelstrasse 43, 3012 Bern, Switzerland; 2 Department of Sociology, Ludwig-Maximilians-Universität München, Munich, Germany; 3 Centre for Infectious Disease Epidemiology & Research, School of Public Health, University of Cape Town, Cape Town, South Africa; 4 Medscheme, Cape Town, South Africa; 5 Department of Nuclear Medicine, University of Pretoria, Pretoria, South Africa; 6 Steve Biko Academic Hospital, Pretoria, South Africa; 7 Department of Infectious Diseases, Bern University Hospital, University of Bern, Bern, Switzerland; 8 Graduate School of Health Sciences, University of Bern, Bern, Switzerland; 9 Department of Psychiatry and New York State Psychiatric Institute, Columbia University, New York, NY USA; 10 Population Health Sciences, Bristol Medical School, University of Bristol, Bristol, UK; 11 Division of Clinical Pharmacology, Department of Medicine, University of Cape Town, Cape Town, South Africa; 12 HIV Mental Health Research Unit, Neuroscience Institute, University of Cape Town, Cape Town, South Africa; 13 Division of Neuropsychiatry, Department of Psychiatry and Mental Health, Faculty of Health Sciences, University of Cape Town, Cape Town, South Africa

**Keywords:** Mental disorders, HIV, Antiretroviral therapy, Adherence, Viral suppression, South Africa

## Abstract

We followed adolescents and adults living with HIV aged older than 15 years who enrolled in a South African private-sector HIV programme to examine adherence and viral non-suppression (viral load > 400 copies/mL) of participants with (20,743, 38%) and without (33,635, 62%) mental health diagnoses. Mental health diagnoses were associated with unfavourable adherence patterns. The risk of viral non-suppression was higher among patients with organic mental disorders [adjusted risk ratio (aRR) 1.55, 95% confidence interval (CI) 1.22–1.96], substance use disorders (aRR 1.53, 95% CI 1.19–1.97), serious mental disorders (aRR 1.30, 95% CI 1.09–1.54), and depression (aRR 1.19, 95% CI 1.10–1.28) when compared with patients without mental health diagnoses. The risk of viral non-suppression was also higher among males, adolescents (15–19 years), and young adults (20–24 years). Our study highlights the need for psychosocial interventions to improve HIV treatment outcomes—particularly of adolescents and young adults—and supports strengthening mental health services in HIV treatment programmes.

**Supplementary Information:**

The online version contains supplementary material available at 10.1007/s10461-022-03916-x.

## Resumen

Monitoreamos adolescentes y adultos mayores de 15 años que viven con VIH y que están registrados en un programa privado Surafricano para el tratamiento del VIH. Nuestro propósito fue examinar adherencia a los medicamentos y supresión viral (carga viral < 400 copias/mL) en los participantes con (20,743, 38%) y sin (33,635, 62%) diagnósticos de salud mental. Los diagnósticos de salud mental estuvieron asociados con patrones de adherencia desfavorables. Comparados con pacientes sin diagnósticos de salud mental, el riesgo de no supresión viral fue más alto entre pacientes con desórdenes mentales orgánicos [riesgo relativo ajustado (aRR) 1.55, 95% intervalo de confidencia (CI) 1.22–1.96], desórdenes en el uso de sustancias (aRR 1.53, 95% CI 1.19–1.97), desórdenes mentales serios (aRR 1.30, 95% CI 1.09–1.54), y depresión (aRR 1.19, 95% CI 1.10–1.28). El riesgo de no supresión viral también fue más alto en hombres que en mujeres, en adolescentes (15–19 años), y en adultos jóvenes. Nuestro estudio resalta la necesidad de intervenciones psicosociales para mejorar los resultados del tratamiento contra el VIH –particularmente en adolescentes y adultos jóvenes—, y respalda el fortalecimiento de servicios de salud mental como parte de los programas para el tratamiento del VIH.

## Introduction

South Africa has the largest HIV epidemic globally with approximately 7.8 million people living with HIV and over 5 million people receiving antiretroviral therapy (ART) [1]. Widespread access to ART has improved the life expectancy of people living with HIV [2], yet long-term effectiveness of ART depends on lifelong retention in HIV care and high levels of ART adherence for achieving long-term viral suppression (HIV RNA < 200 copies/mL) [3, 4]. A recent study estimated that 75% adherence to integrase inhibitor-based regimens and 78% adherence to nonnucleoside reverse transcriptase inhibitor-based regimens are required to achieve viral suppression (HIV RNA < 200 copies/mL) in 90% of viral load tests [5].

Mental health and substance use disorders are highly prevalent among people living with HIV [6–9]. Studies have consistently identified higher rates of mental health and substance use disorders experienced by people living with HIV when compared with those in the general population [10]. Among people living with HIV in sub-Saharan Africa, the prevalence of alcohol use disorders ranges from 7 to 31% [8] and the prevalence of depression is estimated at 15% [6], while anxiety disorders among people living with HIV in developing countries is estimated at 23% [7]. The higher prevalence of mental disorders among people living with HIV might be a consequence of both higher rates of HIV acquisition among people with mental disorders due to increased sexual risk behaviour [11, 12] and a greatly increased incidence of mental disorders among people living with HIV due to various biological and psycho-social mechanisms [13].

The co-occurrence of mental illness and HIV poses challenges when treating people living with HIV. Mental disorders are associated with poor HIV treatment outcomes, including low adherence [8, 14–16], poor virological outcomes [17–20], poor retention in HIV care [20, 21], and increased mortality [20]. Estimates on associations between mental health disorders and HIV treatment outcomes vary widely, likely due to methodological limitations of previous studies, such as cross-sectional study designs, small sample sizes, and self-reported mental health and adherence measures. Self-reported mental health screening tools often have imperfect specificity and low positive predictive values for mental health diagnoses according to diagnostic criteria [22, 23]. Self-reported adherence measures are prone to overreporting and poorly predictive of viral suppression [24–26]. In contrast, objective pharmacy-based adherence consistently predicts virological and other clinical outcomes [24, 26, 27].

Using mental health diagnoses from reimbursement claims, an objective pharmacy-based adherence measure, and routine viral load data from a large cohort of adolescents and adults who enrolled in a private sector HIV management programme in South Africa, we examined associations between mental health diagnoses, ART adherence, and viral suppression.

## Methods

### Study Design

We followed a cohort of adolescents and adults living with HIV who enrolled in the Aid for AIDS (AfA) HIV management programme from their first documented ART use (baseline) to the end of insurance coverage, death, or database closure, whichever occurred first. AfA received ethical approval to contribute data to the International epidemiology Databases to Evaluate AIDS (IeDEA) [28]. The Human Research Ethics Committee of the University of Cape Town, South Africa and the Cantonal Ethics Committee Bern, Switzerland authorised the analysis of the database. Beneficiaries of the medical insurance scheme or their guardians provided consent for their data to be used in research.

### Setting

The AfA programme is a private sector HIV management programme for insured people living with HIV and their families in South Africa. Medical insurance plans provide unlimited HIV/AIDS benefits for patients in the AfA programme. For single adults, medical insurance plans start from approximately 1300 ZAR—equivalent to 60 times the hourly minimum wage in South Africa. In 2018, 15% of the population were covered by private sector medical insurance [29]. Private medical practitioners and specialists treat patients with HIV according to national treatment guidelines [30]. General practitioners, psychiatrists, psychologists, and private inpatient mental health facilities provide mental health care. We did not include patients accessing HIV care in the public sector in our study.

### Eligibility

Adolescents and adults living with HIV aged 15 years or older covered by insurance from a large South African medical insurance scheme at any point between January 1, 2011 and June 30, 2020 who received ART for at least 6 months and enrolled in the AfA programme were eligible for inclusion. We excluded individuals missing sex or date of birth information. We also excluded patients with fewer than 1 year of follow-up from our analysis of factors associated with adherence; people with less than 3 years of follow-up from our adherence trajectory analysis; and patients without viral load measurement from our analysis of viral suppression (Fig. S1).

### Data

We extracted demographic and laboratory data and reimbursement claims from the IeDEA database [28]. Pharmacy claims contained information about active drug ingredients coded according to the Anatomical Therapeutic Chemical (ATC) classification system [31], drug strength, dispensed amount, and dispensed date. Outpatient and hospitalisation claims contained International Classification of Diseases, 10th Revision (ICD-10) diagnoses [32]. Laboratory data contained HIV viral load and CD4 cell counts. Claims data were available from January 1, 2011 to June 30, 2020; and laboratory data from January 1, 2016 to June 30, 2020.

### Outcomes

We defined viral non-suppression (VNS) as an HIV viral load ≥ 400 copies/mL and explored thresholds of 100 copies/mL and 1000 copies/mL in sensitivity analyses. We assessed adherence based on pharmacy claims for antiretroviral medication (ATC codes J05AR, J05AG, J05AE, J05AJ, or J05AX). We calculated the duration of each claim by dividing the dispensed amount of pills by the assumed average maintenance dose for adults per WHO’s defined daily doses [33]. Our adherence definition allowed for stockpiling of medication. Participants refilling their prescriptions early could carry over any remaining pills into subsequent observation periods. We calculated participant continuous medication availability (CMA) in two steps [34, 35]. First, we assigned the mean adherence value of an interval between two consecutive refills—or between the last refill and the end of patient follow-up—to each day of the interval by dividing the number of days covered by sufficient drug supply during the interval and the number of days of the interval. Second, we split participant follow-up time into consecutive 1, 3, 6, and 12-month intervals and averaged daily mean adherence values over each interval. We defined non-adherence as CMA values below 80% and explored thresholds of 70% and 90% in sensitivity analyses.

### Exposures

We assessed mental health based on ICD-10 diagnoses from outpatient and hospital claims. We considered diagnoses for any mental disorders in the ICD-10 range F00–F99. We grouped mental disorders into organic mental disorders (ICD-10 codes F00–09); substance use disorders (F10–F19); serious mental disorders (F20–F29 and F31); depression (F32, F33, and F34.1); anxiety (F40–F48); or other mental disorders (F30, F34.0, F34.8, F34.9, F50–F99). In sensitivity analyses, we considered participants diagnosed if they had received at least two diagnoses on different dates. We grouped age ranges into seven categories (15̵–19, 20̵–24, 25–34, 35–44, 45–54, 55–64, and ≥ 65 years).

### Statistical Analysis

Using summary statistics, we described patient characteristics by mental health status. To validate CMA measurements, we estimated true-positive rate, false-positive rate, and area under the curve (AUC) of the CMA at 1, 3, 6, and 12 months before viral load tests for predicting VNS. We estimated these measures using receiver operating characteristic (ROC) regression models with probit link by maximum likelihood estimation because ROC regression allowed adjustments for clustering patient-level data.

We estimated unadjusted and adjusted risk ratios (RR) for factors associated with non-adherence and VNS using mixed-effects Poisson regression models with robust standard errors and a random intercept at patient-level [36, 37]. First, we estimated RRs for each group of mental health diagnoses, adjusting for age, sex, and year since baseline. We rounded year since baseline to the next integer and modelled as a categorical variable. We modelled mental health diagnoses, age, and years since baseline as time-varying covariates. Second, we estimated RRs for each group of mental health diagnoses, adjusting for age, sex, years since baseline, and psychiatric comorbidity. Next, we adjusted RRs for associations between mental health diagnoses and VNS for age, sex, years since baseline, and CMA. Finally, we estimated and plotted adjusted model predictions for outcomes at 2 years after baseline for participants with and without mental health diagnoses by age and sex. We give more details on statistical methods in the appendix (Text S1).

To identify persons with similar adherence trajectories, we performed a longitudinal trajectory analysis using the R package kml [38]. The package implements a k-means expectation–maximization algorithm to cluster observations with homogeneous longitudinal trajectories into distinct groups. In this analysis, we modelled participants’ 3-monthly CMA scores as continuous outcomes. We analysed adherence trajectories over 5 years from baseline. We imputed missing CMA scores for participants with less than 5 years of follow-up based on participants’ trajectory means [38]. We ran the algorithm five times to identify two to six adherence groups and chose the optimal group size based on clinical relevance and Calinski–Harabatz’s and Ray–Turi’s criteria [38]. Based on our visual inspection of mean CMA group plots, we labelled adherence patterns as either ‘continuous high adherence,’ ‘decreasing adherence,’ ‘increasing adherence,’ or ‘continuous non-adherence.’

Finally, we performed a multinomial logistic regression analysis to examine factors associated with classification with an adherence group. The regression model included a categorical variable for age, binary variables for sex and mental health diagnosis at baseline, and an interaction term between age and sex. We estimated and plotted the probability of group affiliation by sex, age, and mental health status.

We performed statistical analyses in Stata (Version 16) and R (R version 3.6.3).

## Results

### Characteristics of Participants

We followed 54,378 adolescents and adults with HIV for a median duration of 3.5 years (IQR 1.9–6.4). The median age of participants at baseline was 40.1 years (SD 9.9), and most were female (59%). At the end of follow-up, 38% of participants had been diagnosed with at least one mental disorder. Anxiety (26%) and depression (20%) were the most prevalent mental health diagnoses (Table 1). Mental health diagnoses were more prevalent among females (43%) than males (32%). The prevalence of mental health diagnoses peaked among males (34%) and females (48%) aged 45–54 years (Table S1).

**Table 1:** Characteristics of participants by mental health status at the end of follow-up

	No mental health diagnosis	Mental health diagnosis	Total
	N = 33,635 (61.9)	N = 20,743 (38.1)	N = 54,378 (100.0)
Age, years			
15–19	828 (2.5)	385 (1.9)	1213 (2.2)
20–24	698 (2.1)	329 (1.6)	1027 (1.9)
25–34	8524 (25.3)	5498 (26.5)	14,022 (25.8)
35–44	12,474 (37.1)	8259 (39.8)	20,733 (38.1)
45–54	8117 (24.1)	4898 (23.6)	13,015 (23.9)
55–64	2720 (8.1)	1267 (6.1)	3987 (7.3)
65+	274 (0.8)	107 (0.5)	381 (0.7)
Mean (SD)	40.2 (10.1)	39.8 (9.4)	40.1 (9.9)
Sex			
Male	15,219 (45.2)	7131 (34.4)	22,350 (41.1)
Female	18,416 (54.8)	13,612 (65.6)	32,028 (58.9)
ART regimen at baseline			
NNRTI-based	29,650 (88.2)	18,118 (87.3)	47,768 (87.8)
II-based	200 (0.6)	131 (0.6)	331 (0.6)
PI-based	3785 (11.3)	2494 (12.0)	6279 (11.5)
Follow-up time, years			
Median (IQR)	3.0 (1.5–5.4)	4.9 (2.8–7.6)	3.5 (1.9–6.4)
Mental health diagnoses			
Organic mental disorder	0 (0.0)	488 (2.4)	488 (0.9)
Substance use disorder	0 (0.0)	429 (2.1)	429 (0.8)
Serious mental disorder	0 (0.0)	1235 (6.0)	1235 (2.3)
Depression	0 (0.0)	11,000 (53.0)	11,000 (20.2)
Anxiety	0 (0.0)	14,248 (68.7)	14,248 (26.2)
Other mental disorders	0 (0.0)	3446 (16.6)	3446 (6.3)

Data are number of participants and percentages if not stated otherwise

*SD* standard deviation, *IQR* interquartile range, *ART* antiretroviral therapy, *NNRTI* non-nucleoside reverse transcriptase inhibitors, *II* integrase inhibitor, *PI* protease inhibitor

### Validation of CMA

CMA measured over 12 months before viral load testing had an AUC of 0.82 (95% CI 0.81–0.83) for predicting VNS. CMA measured over a shorter duration had a slightly lower AUC (Fig. S2).

### Mental Health and Adherence

**Fig. 1:**
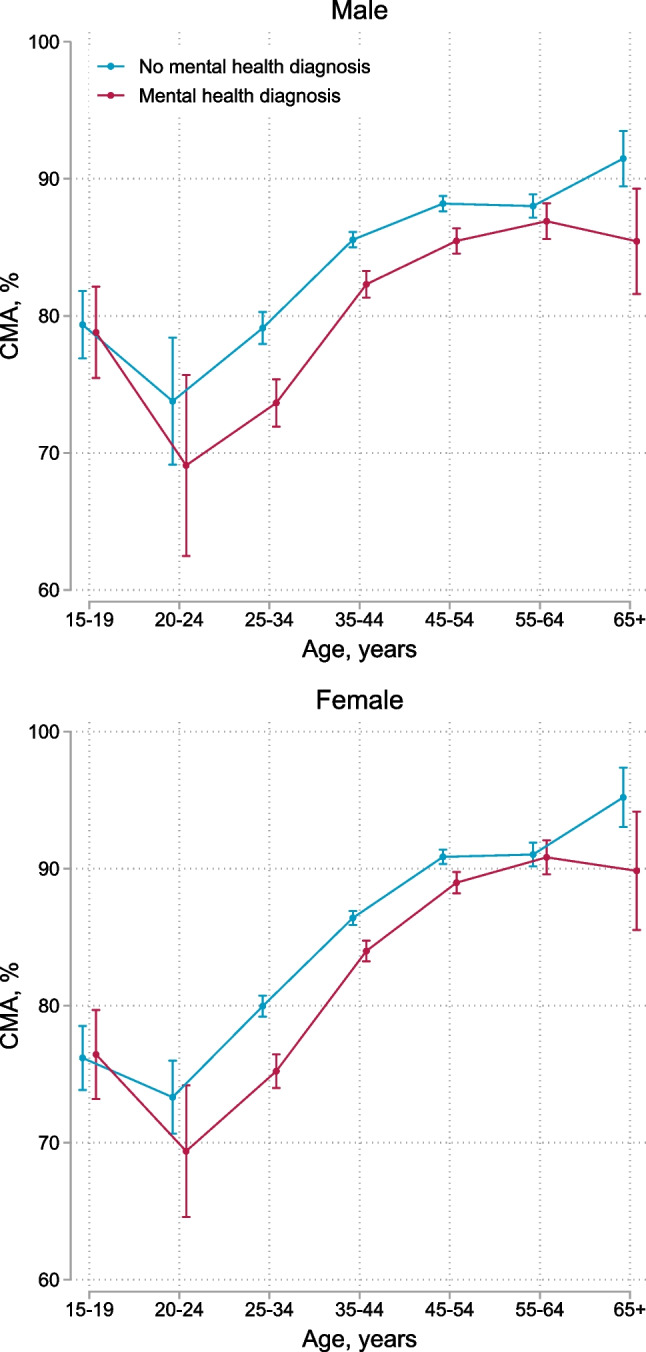
Cumulative medication availability (CMA) in the 2nd year after baseline comparing participants with and without mental health diagnoses by age group and sex. Error bars represent 95% confidence intervals for means and proportions. N = 48,645

Figure 1 shows the estimated mean CMA in the 2nd year after baseline among participants with and without mental health diagnosis by sex and age. Mean CMA ranged from 69% (95% CI 62–76) among males aged 20–24 years who received mental health diagnoses to 95% (95% CI 93–97) among females ≥ 65 years who had not received mental health diagnoses. Females with and without mental health diagnoses aged 45 years and older had a higher mean CMA than males (Fig. S3). We observed no sex differences in younger age groups. In a model adjusted for mental health diagnosis of any mental disorder, age, sex, and year since baseline, participants with a mental health diagnosis (aRR 1.21, 95% CI 1.18–1.25), males (aRR 1.25, 95% CI 1.16–1.34), and younger age groups were at increased risk of non-adherence (CMA < 80%) (Table 2). Specifically, adolescents aged 15–19 years had 35% (aRR 1.35, 95% CI 1.24–1.46) higher risk of non-adherence (CMA < 80%) when compared with adults aged 25–34 years, while participants aged 20–24 years had 40% (aRR 1.40, 95% 1.27–1.54) higher risk (Table 2). Organic mental disorders (aRR 1.17, 95% CI 1.00–1.38), substance use disorders (aRR1.41, 95% CI 1.24–1.62), depression (aRR 1.14, 95% CI 1.10–1.18), and anxiety (aRR 1.17, 95% CI 1.13–1.21) were associated with non-adherence in multivariable analysis adjusted for age, sex, year since baseline, and comorbid mental health diagnoses (Table 2).

**Table 2:** Unadjusted and adjusted risk ratios for factors associated with non-adherence (CMA < 80%)

	Unadjusted risk ratio (95% CI)	Adjusted risk ratio^a^ (95% CI)	Adjusted risk ratio^b^ (95% CI)
Mental health diagnoses			
No mental health diagnosis	1.00	1.00	1.00
Organic mental disorder	1.42 (1.21–1.66)	1.31 (1.12–1.54)	1.17 (1.00–1.38)
Substance use disorder	1.87 (1.64–2.13)	1.60 (1.41–1.83)	1.41 (1.24–1.62)
Serious mental disorder	1.32 (1.19–1.46)	1.23 (1.11–1.36)	1.06 (0.95–1.18)
Depression	1.32 (1.28–1.37)	1.21 (1.17–1.25)	1.14 (1.10–1.18)
Anxiety	1.33 (1.29–1.37)	1.22 (1.18–1.26)	1.17 (1.13–1.21)
Other mental disorders	1.23 (1.15–1.31)	1.10 (1.03–1.17)	1.02 (0.96–1.09)
Any mental disorder	1.33 (1.29–1.37)	1.21 (1.18–1.25)	
Age, years			
15–19	1.38 (1.27–1.49)	1.35 (1.24–1.46)	1.36 (1.25–1.48)
20–24	1.29 (1.19–1.40)	1.40 (1.27–1.54)	1.40 (1.27–1.54)
25–34	1.00	1.00	1.00
35–44	0.79 (0.76–0.81)	0.72 (0.69–0.74)	0.72 (0.69–0.74)
45–54	0.63 (0.61–0.66)	0.55 (0.52–0.57)	0.55 (0.52–0.57)
55–64	0.66 (0.62–0.70)	0.54 (0.51–0.57)	0.54 (0.51–0.57)
65+	0.48 (0.39–0.58)	0.37 (0.31–0.45)	0.37 (0.31–0.46)
Sex			
Male	1.11 (1.07–1.14)	1.25 (1.16–1.34)	1.25 (1.16–1.34)
Female	1.00	1.00	1.00

*CMA* cumulative medication availability, *CI* confidence interval

^a^Risk ratios for each group of mental health diagnoses were adjusted for years since baseline, age, and sex

^b^Risk ratios adjusted for years since baseline, age, sex, organic mental disorders, substance use disorders, serious mental disorders, depression, anxiety, and other mental disorders

**Fig. 2:**
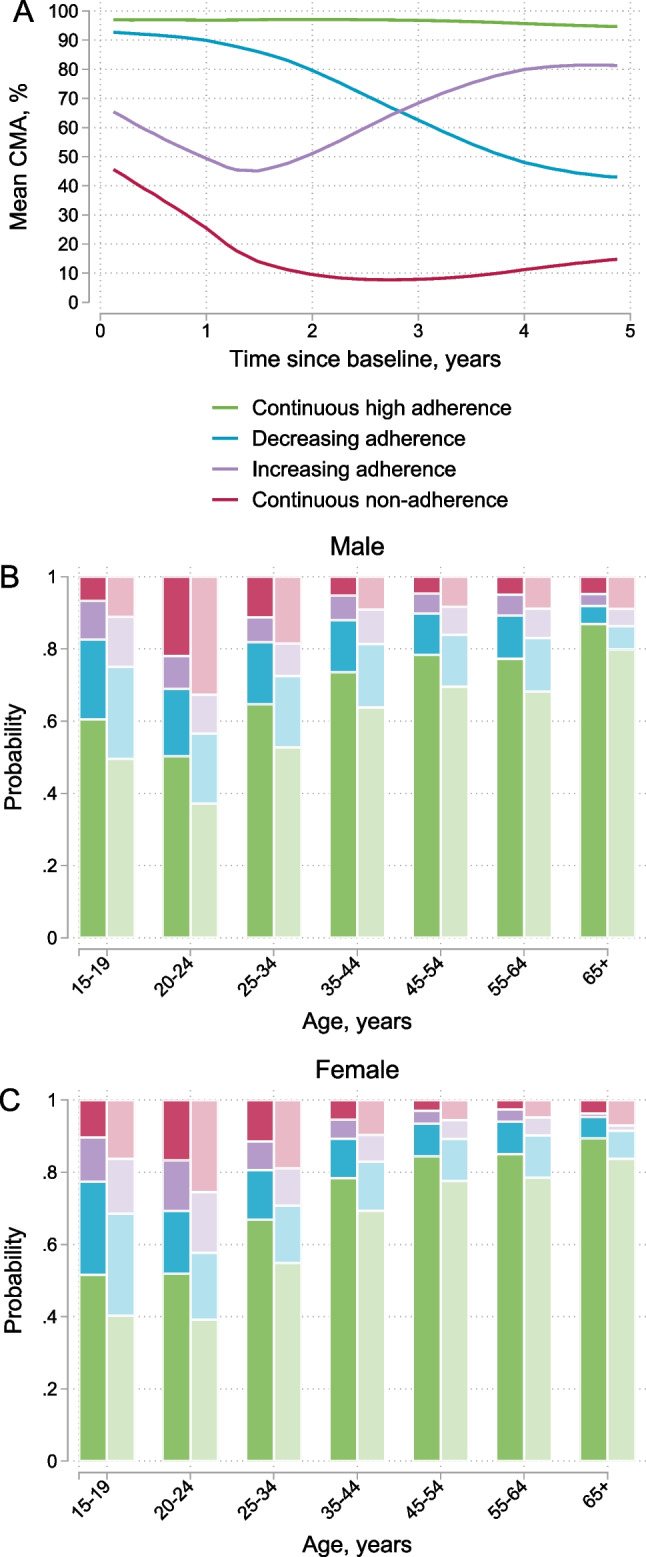
Adherence trajectories and the probability of being in each adherence group by sex, age and mental health diagnosis at baseline. **A** Shows the mean cumulative medication adherence (CMA) for the four groups identified in the longitudinal adherence trajectory analysis. **B**, **C** Shows the probability of being in each group by sex, age, and mental health diagnosis at baseline. Darker colours represent persons who did not received mental health diagnoses (ICD10 F00–F99) before or at baseline and lighter colours for those who received mental health diagnoses. We included participants with at least 3 years of follow-up in the analysis (N = 32,254)

In the adherence trajectory analysis, we identified four distinct longitudinal adherence trajectories. We show estimated mean CMA for each group in Fig. 2A. Most participants had continuously high adherence (73%, 23,686/32,254), some participants decreasing (13%, 4152) or increasing (6%, 2073) adherence, and some participants continuous non-adherence (7%, 2343). Participants who received mental health diagnoses at baseline were more likely to have decreasing adherence (aRR 1.41, 95% CI 1.28–1.55), increasing adherence (aRR 1.59, 95% 1.41–1.79), or continuous non-adherence (aRR 2.02, 95% 1.81–2.25) when compared with participants who did not receive mental health diagnoses. Younger age was the strongest predictor of suboptimal adherence patterns (Table 3). For example, young adults aged 20–24 years had more than twice the risk of continuous non-adherence (aRR 2.18, 95% CI 1.54–3.08) when compared with slightly older participants aged 25–34 (Table 3).

**Table 3:** Risk ratios for factors associated with decreasing adherence, increasing adherence, or continuous non-adherence compared with continuously high adherence

	Decreasing adherence	Increasing adherence	Continuous non-adherence
	aRR (95% CI)	aRR (95% CI)	aRR (95% CI)
Mental health diagnosis at baseline			
No mental health diagnosis	1.00	1.00	1.00
Mental health diagnosis	1.41 (1.28–1.55)	1.59 (1.41–1.79)	2.02 (1.81–2.25)
Age, years			
15–19	1.83 (1.48–2.25)	1.80 (1.37–2.38)	0.87 (0.64–1.18)
20–24	1.51 (1.05–2.18)	1.95 (1.22–3.13)	2.18 (1.54–3.08)
25–34	1.00	1.00	1.00
35–44	0.71 (0.65–0.77)	0.70 (0.62–0.79)	0.41 (0.37–0.45)
45–54	0.54 (0.48–0.59)	0.49 (0.42–0.56)	0.27 (0.23–0.30)
55–64	0.55 (0.47–0.64)	0.49 (0.39–0.61)	0.25 (0.20–0.33)
65+	0.26 (0.15–0.47)	0.18 (0.06–0.54)	0.28 (0.15–0.54)
Sex			
Male	1.14 (0.93–1.41)	1.31 (0.92–1.88)	1.21 (0.95–1.53)
Female	1.00	1.00	1.00

*aRR* adjusted risk ratios

We show predicted probabilities for each adherence group by sex, age, and mental health diagnoses at baseline in Fig. 2B, C. Suboptimal adherence patterns were more prevalent among younger age groups and participants with mental health diagnoses than in older age groups and those without mental health diagnoses. For example, males aged 20–24 years with mental health diagnoses had the highest risk of being continuously non-adherent (33%, 95% CI 20–46), whereas females aged 55–64 years without mental health diagnoses had the lowest risk (3%, 95% CI 2–4). We show 95% CIs for predicted probabilities in Table S2.

### Mental Health and Viral Suppression

**Fig. 3:**
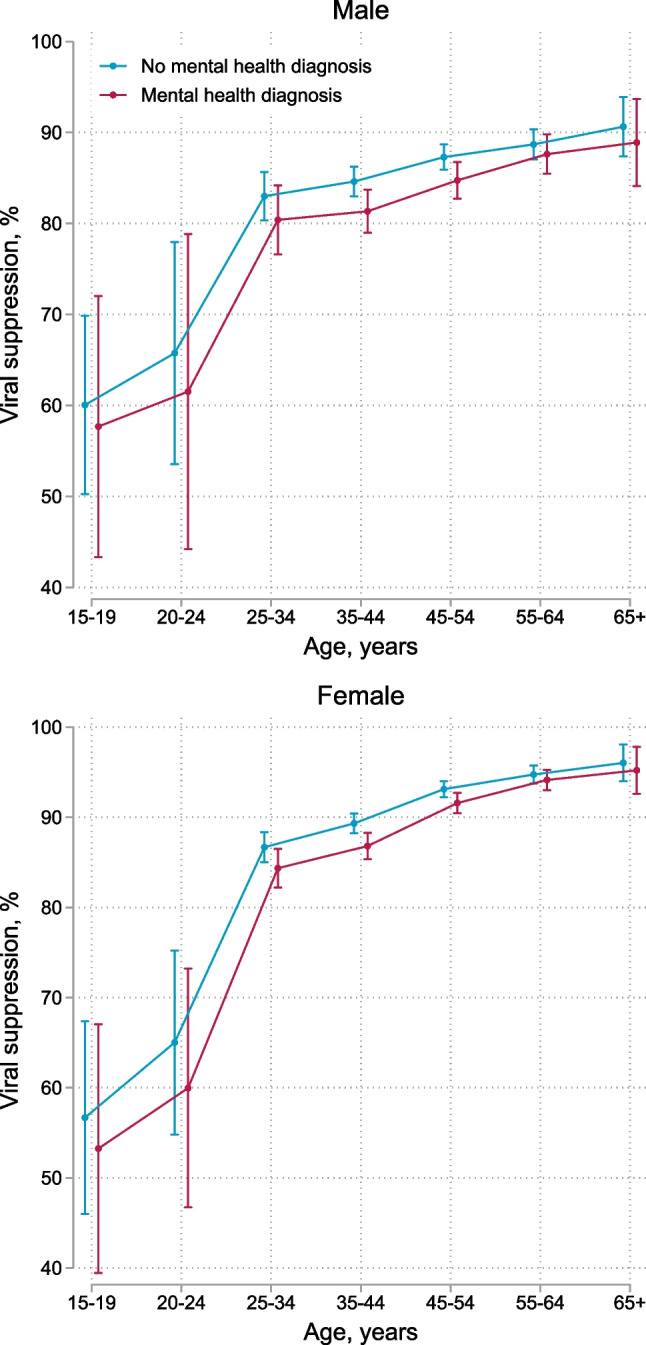
Viral suppression (viral load < 400 copies/mL) at 2 years after baseline comparing participants with and without mental health diagnoses by age and sex. Error bars represent 95% confidence intervals for means and proportions. N = 28,785

Overall, 90% (71,433/79,463) of recorded viral load measurements were below the viral load threshold of 400 copies/mL. We present viral suppression (viral load < 400 copies/mL) rates 2 years after baseline by sex, age, and mental health status in Fig. 3. Viral suppression rates ranged from 53% (95% CI 39–67) among adolescent females aged 15–19 with mental health diagnoses to 96% (95% CI 94–98) among females aged 65 years or older without mental health diagnoses. Viral suppression rates increased with increasing age, yet were lower among participants with mental health diagnoses. Females older than age 25 had higher suppression rates than males; adolescent females and young females under age 25 had lower suppression rates than their male counterparts (Fig. S4). We show numerical values for viral suppression rates by sex, age group, and mental health status at 2 years after baseline for all three viral load thresholds (100, 400, and 1000 copies/mL) in Table S3. In all adjusted models, male sex and younger age were strongly associated with VNS (Table 4). In models adjusted for age, sex, and years since baseline, VNS was associated with organic mental disorders (aRR 1.78, 95% CI 1.41–2.24), substance use disorders (aRR 1.82 95% CI 1.42–2.34), serious mental disorders (aRR 1.54, 95% CI 1.30–1.82), depression (aRR 1.26, 95% CI 1.17–1.35), anxiety (aRR 1.11, 95% CI 1.04–1.19), and other mental disorders (aRR 1.10, 95% CI 0.98–1.23). In models adjusted for age, sex, and psychiatric comorbidity, associations between all mental health diagnoses and VNS were attenuated; diagnoses of anxiety or other mental disorders were no longer associated (Table 4). The association between substance use disorders and VNS was fully mediated by CMA. Associations between other mental health diagnoses and VNS were partially mediated by CMA (Table 4).

**Table 4:** Unadjusted and adjusted risk ratios for factors associated with viral non-suppression (viral load ≥ 400 copies/mL)

	RR (95% CI)	aRR (95% CI)^a^	aRR (95% CI)^b^	aRR (95% CI)^c^
Mental health diagnoses				
No mental health diagnosis	1.00	1.00	1.00	1.00
Organic mental disorder	1.70 (1.34–2.15)	1.78 (1.41–2.24)	1.55 (1.22–1.96)	1.34 (1.09–1.66)
Substance use disorder	2.03 (1.58–2.60)	1.82 (1.42–2.34)	1.53 (1.19–1.97)	1.03 (0.83–1.27)
Serious mental disorder	1.42 (1.20–1.68)	1.54 (1.30–1.82)	1.30 (1.09–1.54)	1.27 (1.09–1.48)
Depression	1.17 (1.09–1.26)	1.26 (1.17–1.35)	1.19 (1.10–1.28)	1.11 (1.04–1.19)
Anxiety	1.03 (0.97–1.10)	1.11 (1.04–1.19)	1.04 (0.97–1.11)	1.00 (0.95–1.07)
Other mental disorders	1.08 (0.97–1.22)	1.10 (0.98–1.23)	1.01 (0.90–1.14)	1.06 (0.96–1.17)
Any mental disorder	1.08 (1.02–1.15)	1.16 (1.09–1.24)		
Age, years				
15–19	2.85 (2.40–3.38)	2.71 (2.28–3.22)	2.72 (2.29–3.24)	2.52 (2.14–2.96)
20–24	2.42 (1.97–2.97)	2.29 (1.84–2.85)	2.29 (1.83–2.85)	1.93 (1.55–2.42)
25–34	1.00	1.00	1.00	1.00
35–44	0.89 (0.82–0.97)	0.87 (0.79–0.95)	0.87 (0.80–0.95)	0.96 (0.89–1.04)
45–54	0.67 (0.61–0.74)	0.63 (0.57–0.70)	0.64 (0.57–0.70)	0.82 (0.75–0.89)
55–64	0.56 (0.50–0.63)	0.50 (0.44–0.57)	0.50 (0.44–0.57)	0.68 (0.61–0.77)
65+	0.47 (0.36–0.60)	0.41 (0.31–0.54)	0.41 (0.31–0.54)	0.62 (0.48–0.80)
Sex				
Male	1.42 (1.33–1.52)	1.47 (1.31–1.66)	1.48 (1.31–1.67)	1.34 (1.20–1.50)
Female	1.00	1.00	1.00	1.00
CMA, %				
100	1.00			1.00
90–99	1.55 (1.43–1.68)			1.48 (1.37–1.61)
80–89	2.26 (2.07–2.46)			2.14 (1.97–2.34)
70–79	3.00 (2.73–3.30)			2.83 (2.57–3.11)
60–69	3.58 (3.23–3.96)			3.39 (3.07–3.76)
50–59	4.54 (4.09–5.04)			4.21 (3.79–4.67)
40–49	5.41 (4.85–6.04)			5.09 (4.56–5.69)
30–39	6.32 (5.67–7.03)			5.97 (5.36–6.64)
20–29	7.24 (6.59–7.96)			6.73 (6.13–7.40)
10–19	9.96 (9.24–10.74)			9.40 (8.71–10.14)
0–9	15.89 (14.88–16.98)			15.20 (14.20–16.27)

*RR* risk ratio, *aRR* adjusted risk ratio, *CI* confidence interval, *CMA* cumulative medication availability

^a^Risk ratios for each group of mental health diagnoses were adjusted for years since baseline, age, and sex

^b^Risk ratios were adjusted for years since baseline, age, sex, organic mental disorders, substance use disorders, serious mental disorders, depression, anxiety and other mental disorders

^c^Risk ratios were adjusted for years since baseline, age, sex, organic mental disorders, substance use disorders, serious mental disorders, depression, anxiety, other mental disorders and CMA

### Sensitivity Analyses

Our conclusions were robust in sensitivity analyses with different thresholds for non-adherence (CMA < 70% and < 80%) and VNS (viral load > 100 copies/mL and > 1000 copies/mL) or when using an alternative exposure definition requiring participant diagnoses at least twice on different dates (Tables S4, S5).

## Discussion

In our study cohort of privately insured adolescents and adults living with HIV, mental health diagnoses, younger age, and sex were associated with unfavourable adherence patterns and VNS. Among participants with depression and anxiety, the increase in the risk of adverse HIV treatment outcomes was modest. In contrast, participants with serious mental disorders, substance use disorders, and organic mental disorders were at substantially higher risk of VNS and non-adherence than those without mental health diagnoses. Adolescents and young adults with and without mental health diagnoses had low viral suppression rates, whereas older adults generally had high suppression rates, even those with mental health diagnoses. Middle aged males were at higher risk of non-adherence and VNS than females of the same age.

Our results highlight the large burden of mental illness among adolescents and young adults living with HIV in South Africa and underline the need to strengthen mental health care into HIV treatment programs. Over a median follow-up of 3.5 years, 38% of participants enrolled in our study were diagnosed with a mental disorder; 20% with depression; and 26% with anxiety. Systematic reviews of the burden of depression and anxiety among adults living with HIV in low- and middle-income settings produced similar results for depression (15%) and anxiety (23%) [6, 7], indicating high rates of ascertainment of these disorders in the AfA program. By contrast, the low proportion of participants diagnosed with substance use disorders likely reflects the under-ascertainment of prevalent alcohol and substance use disorders [8, 39]. The stigma attached to substance use disorders [40, 41] and health care providers’ limited knowledge of the diagnostic criteria for substance use conditions [42] may contribute to underdiagnoses. Importantly, conclusions regarding access to mental health care cannot be generalised to patients accessing public sector HIV care programs. A previous study showed much lower rates of ascertainment and treatment of mental disorders in the public sector than in the private sector in South Africa [43]. Interventions addressing the high burden of mental illness among people living with HIV are needed, especially in South Africa’s public sector. Routine screening for mental health and substance use disorders [44, 45] and shifting mental health counselling from specialised to non-specialised health workers or trained laypersons are promising approaches for integrating mental health services in primary care HIV care programs in low- and middle-income countries [46–53].

Our study confirms and extends previous findings on associations between mental health and substance use conditions and HIV treatment outcomes. Aligned with previous systematic reviews of studies on adults, we found depression, anxiety, and alcohol and substance use disorders associated with non-adherence. In a meta-analysis of 11 studies from Sub-Saharan Africa, people living with HIV and depression or depressive symptoms had 55% lower odds of achieving optimal ART adherence compared with those without depression or depressive symptoms [8]. These results were confirmed in a meta-analysis of 111 studies conducted in low- and middle-income countries [15]. In another meta-analysis, anxiety symptoms were associated with 59% higher odds of suboptimal ART adherence [14]. Two further systematic reviews reported associations between alcohol use disorders and low adherence to ART [8, 16]. Our study extends previous work by demonstrating that less prevalent yet more serious mental disorders, such as bipolar disorder or schizophrenia, are more strongly associated with non-adherence than common mental disorders. Involving significant others as treatment partners or directly observed therapy are recommended strategies to improve adherence among persons with serious mental illnesses [54]. Substance use disorders were also strongly associated with non-adherence and non-suppressed viral load, suggesting a need for expanding harm reduction programs and adherence interventions [55, 56].

Patients with mental illness should be assessed individually without implicit bias regarding their medication adherence [54]. Although patients diagnosed with mental disorders were at an increased relative risk of VNS, most had good adherence and achieved viral suppression. Therefore, patients with mental illness should be considered eligible for differentiated ART delivery models intended for clinically stable patients [57, 58]. Although some patients receiving care for their mental health benefit from close monitoring and mental health interventions [51, 52], patients with well-controlled mental illness who adhere to ART should have equal access to differentiated service delivery models [58].

Consistent with previous reports [59, 60], adolescents and young adults had poorer HIV treatment outcomes than older adults. In our study, only about 40–60% of participants younger than 25 years had continuously high adherence. The proportion of participants with decreasing adherence was highest among adolescents aged 15–19 years. The decrease in adherence might reflect challenges related to transitioning from paediatric to adolescent care [61]. The risk of continuous non-adherence was highest among young adults aged 20–24 years, peaking at 33% among young males with mental health diagnoses. In adolescents aged 15–19 years, the risk of continuous non-adherence was 7–16%. Continuous non-adherence reflects long treatment interruptions or discontinuation of ART. A multi-cohort study from South African public sector HIV treatment programs reported much higher rates of loss to follow-up (> 60% at 2 years after ART initiation) among this age group [62]. Poor HIV treatment outcomes among adolescents and young adults highlight the need for interventions for improving care outcomes in this age group. A recent meta-analysis found that psychosocial interventions for adolescents and young people living with HIV showed small-to-moderate effects on adherence and viral load [63]. Scaling-up successful interventions for improving HIV outcomes among young people living with HIV should be a priority.

Males older than age 35 were at higher risk of VNS when compared with contemporaneous females. This result aligns with previous studies reporting poorer HIV outcomes among males than females [64]. Sex disparities within HIV outcomes have been attributed to male health care-seeking behaviour arising from harmful masculine norms, higher rates of harmful alcohol and substance use leading to poor ART adherence, and the gendered nature of health services creating health care barriers for males [64–68].

Strengths of our study include the large sample size, allowing for disaggregated analyses of common and less prevalent serious mental disorders by sex and age, and the availability of mental health diagnoses from primary, secondary, and tertiary care. Most previous studies relied on brief screening tools [8, 14, 15] that usually have a high false-positive rate and low positive predictive value for mental health diagnoses [23]. Further strengths of our study include the use of an objective validated adherence measure, the longitudinal study design, and the novel analytic methods used to examine longitudinal adherence patterns.

Our findings should be considered in light of the following limitations. First, we classified participant mental health status based on ICD-10 diagnoses from reimbursement claims; thus, we missed participants with undiagnosed mental disorders. Since patients with mild forms of mental disorders might be less likely diagnosed, those with more severe mental illness might be over-represented in our sample of participants with mental health disorders. When using administrative data, a further limitation is possibly miscoded ICD-10 diagnoses. Nevertheless, mental health diagnoses from administrative data generally have a high positive predictive value for research diagnoses [69], and our conclusions held true when we considered only repeated mental health diagnoses in sensitivity analyses. Second, we used a pharmacy claim-based adherence measure that may overestimate adherence if patients do not take all collected medication or underestimate adherence if drugs are obtained without documentation or from other sources, such as public sector clinics. Despite these limitations, objective pharmacy claim-based measures are considered more reliable than self-reported adherence measures [26] used in most previous studies [8, 14, 15]. In addition, the high accuracy of CMA for predicting HIV viral load validates our adherence measure. Third, because laboratory data were only available from 2016 to 2020, we could only include about half of the participants in analyses of viral suppression and VNS. Furthermore, we acknowledge routinely collected viral load data does not reflect the status of study participants who stopped taking ART and no longer attend clinic appointments. As a result, reported viral suppression rates should be interpreted as suppression rates of patients retained in care. Fourth, our study does not represent the general population of people living with HIV in South Africa. We analysed data from a private sector HIV programme for employed and insured persons. Therefore, our findings cannot be generalised to persons accessing HIV care in the public sector. Persons accessing the public healthcare sector generally have lower socioeconomic status. They might be at higher risk of experiencing poor mental health and HIV treatment outcomes than employed and insured persons accessing private health care services [70].

## Conclusions

Our study confirms high rates of mental illness among people living with HIV in South Africa. Mental health and substance use disorders, younger age, and male sex were associated with poor HIV treatment outcomes. However, most participants with mental illness had good adherence and achieved viral suppression. Our findings highlight the need for psychosocial interventions for improving HIV treatment outcomes—particularly for adolescents and young adults—and strengthening mental health care support in HIV treatment programs. Patients with mental illnesses established on ART and adherent to their medication require equal access to differentiated ART delivery models.

## Supplementary Information

Below is the link to the electronic supplementary material.

Supplementary file1 (DOCX 4440 kb)

## Data Availability

Data were obtained from the International epidemiology Databases to Evaluate AIDS–Southern Africa (IeDEA-SA). Data cannot be made available online because of legal and ethical restrictions. To request data, readers may contact IeDEA-SA for consideration by filling out the online form available at https://www.iedea-sa.org/contact-us/.

## Code Availability

Statistical code is available under https://github.com/AndreasDHaas/MH-CMA-VNS.
