# Supplementary material for: Mental Health, ART Adherence, and Viral Suppression Among Adolescents and Adults Living with HIV in South Africa: A Cohort Study
Source: AIDS Behav. Author manuscript; Available in PMC 2023 Jun 1. (PMC10149479; doi:10.1007/s10461-022-03916-x)
Supplement: 1866133_Sup_Material [file NIHMS1866133-supplement-1866133_Sup_Material.docx]

**APPENDICES**

**Figure S1: Flow diagram of patient eligibility for participation**


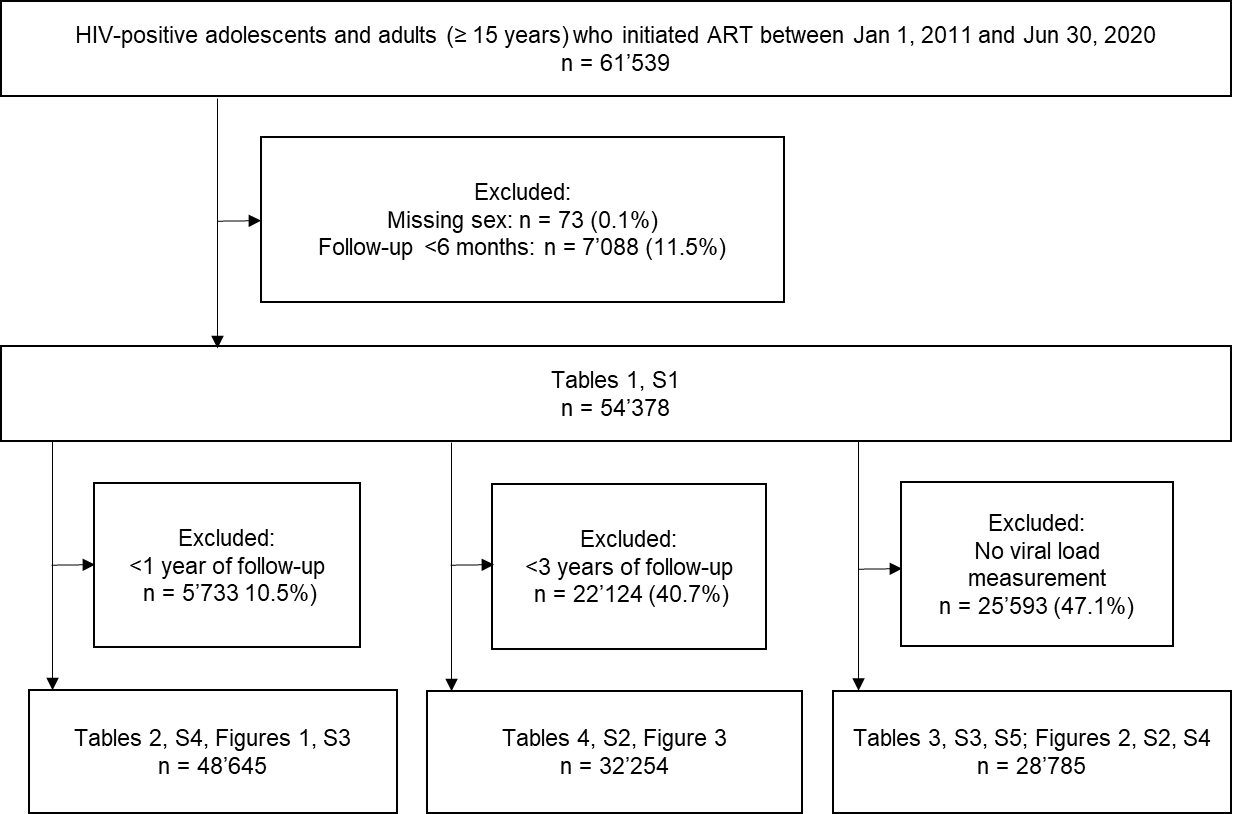


**Figure S2. Receiver operating characteristic (ROC) curves of the accuracy of cumulative medication availability (CMA) for predicting viral non-suppression**

True-positive rate (sensitivity), false-positive rate (1-specificity), and area under the curve (AUC) of cumulative medication availability (CMA) over 1, 3, 6, and 12 months before viral load testing for predicting viral non-suppression (VNS) at a threshold of ≥400 copies/mL. We included 79,463 viral load values from 28,785 participants in the analysis.


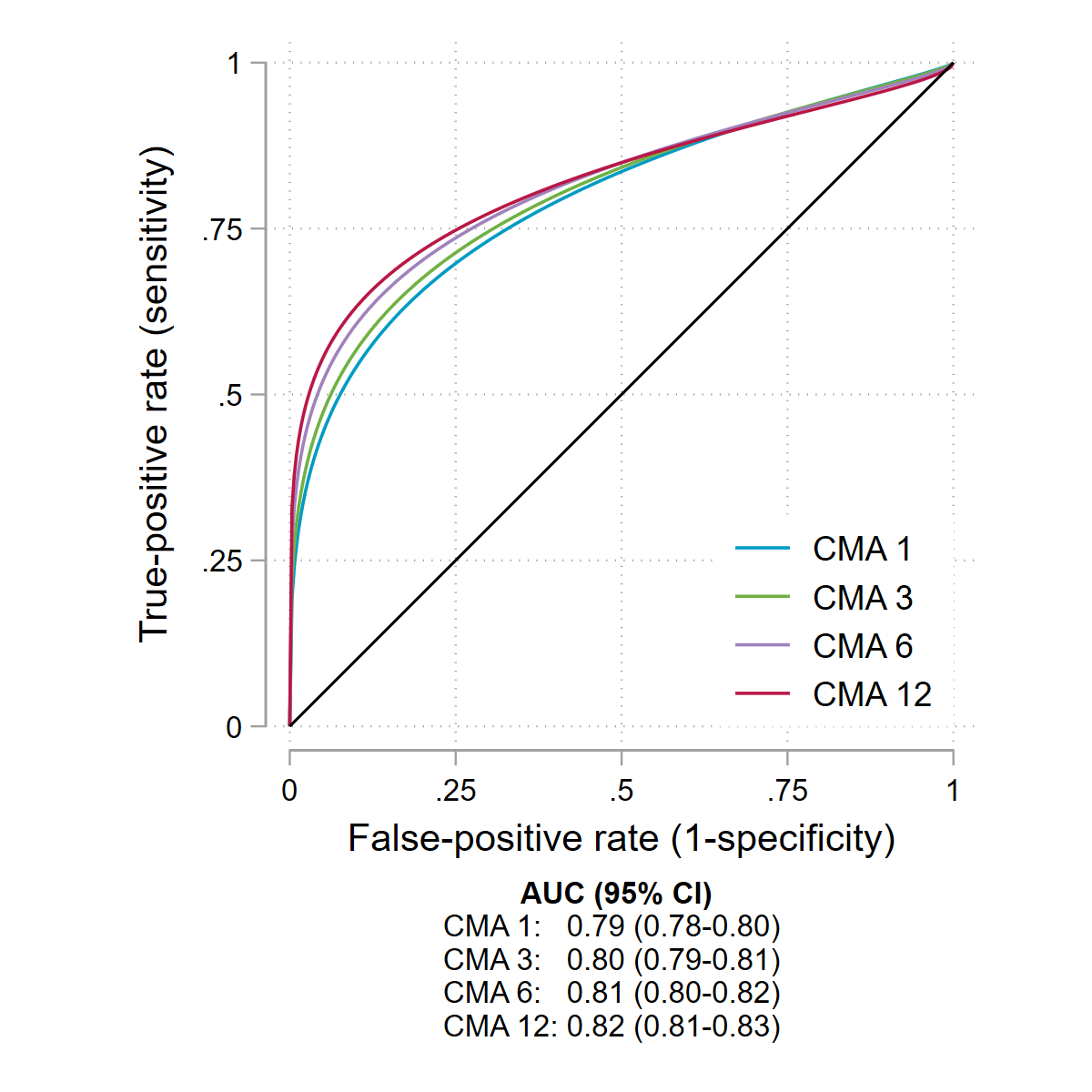


**Figure S3. Cumulative medication availability (CMA) in the second year after baseline comparing males and females by mental health diagnosis and age group**

Error bars represent 95% confidence intervals for means and proportions. N=48,645


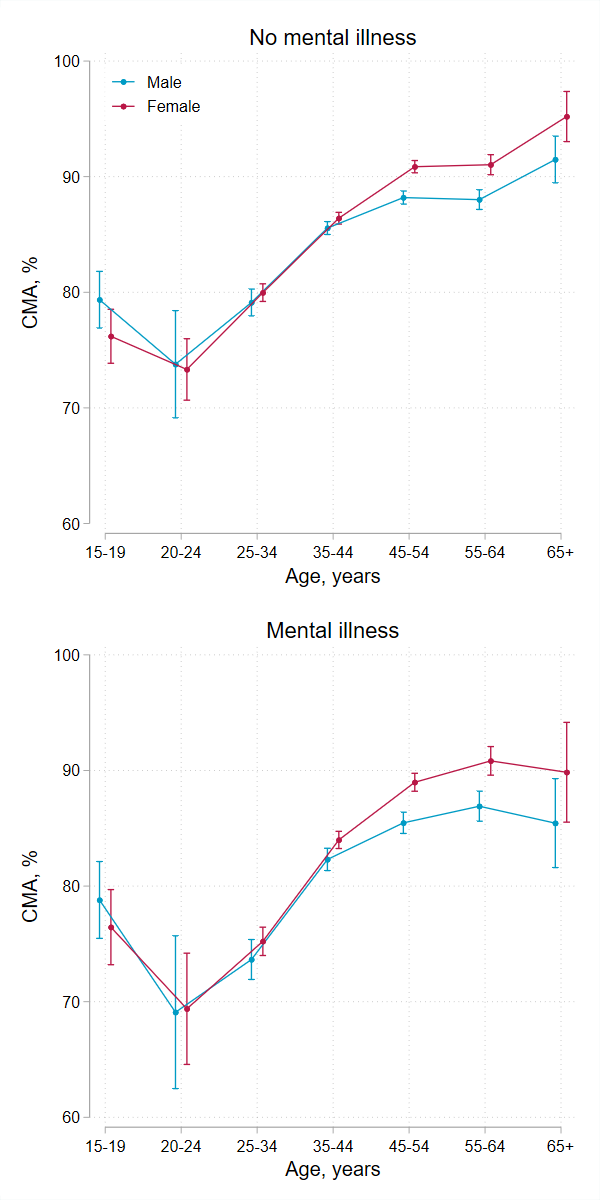


**Figure S4. Viral suppression (viral load <400 copies/mL) at two years after baseline comparing males and females by mental health diagnosis and age group**

Error bars represent 95% confidence intervals for means and proportions. N=28’785


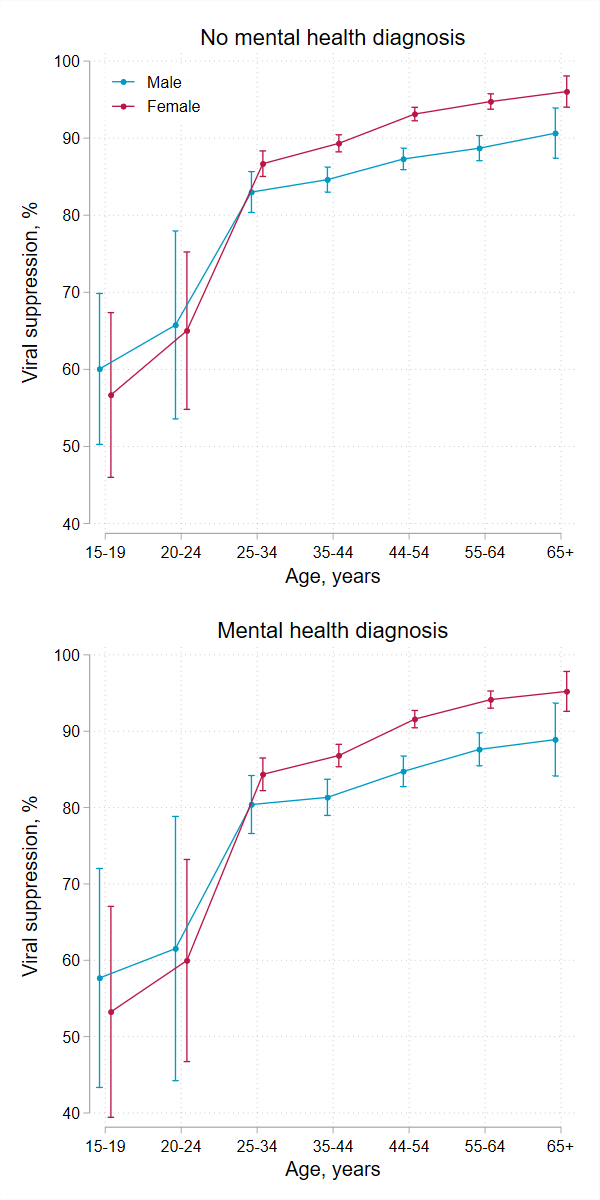


**Table S1: Prevalence of mental health diagnoses at the end of follow-up by sex and age at the end of follow-up**

|  | **Age, years** | | | | | | |  |  |
| --- | --- | --- | --- | --- | --- | --- | --- | --- | --- |
|  | **15–19** | **20–24** | **25–34** | **35–44** | **45–54** | **55–64** | **65+** |  | **Total** |
|  | N=620 (1.1) | N=976 (1.8) | N=7,252 (13.3) | N=19,523 (35.9) | N=16,362 (30.1) | N=8,340 (15.3) | N=1,305 (2.4) |  | N=54,378 (100.0) |
| **Male** | **281 (100.0)** | **293 (100.0)** | **1,827 (100.0)** | **7,062 (100.0)** | **7,709 (100.0)** | **4,394 (100.0)** | **784 (100.0)** |  | **22,350 (100.0)** |
| Mental health diagnosis | 73 (26.0) | 78 (26.6) | 471 (25.8) | 2,217 (31.4) | 2,643 (34.3) | 1,421 (32.3) | 228 (29.1) |  | 7,131 (31.9) |
| Organic mental disorder | 2 (0.7) | 2 (0.7) | 10 (0.5) | 53 (0.8) | 78 (1.0) | 49 (1.1) | 14 (1.8) |  | 208 (0.9) |
| Substance use disorder | 2 (0.7) | 6 (2.0) | 38 (2.1) | 121 (1.7) | 95 (1.2) | 38 (0.9) | 1 (0.1) |  | 301 (1.3) |
| Serious mental disorder | 0 (0.0) | 5 (1.7) | 36 (2.0) | 116 (1.6) | 130 (1.7) | 75 (1.7) | 7 (0.9) |  | 369 (1.7) |
| Depression | 26 (9.3) | 43 (14.7) | 230 (12.6) | 1,083 (15.3) | 1,279 (16.6) | 613 (14.0) | 82 (10.5) |  | 3,356 (15.0) |
| Anxiety | 45 (16.0) | 37 (12.6) | 309 (16.9) | 1,447 (20.5) | 1,735 (22.5) | 898 (20.4) | 132 (16.8) |  | 4,603 (20.6) |
| Other mental disorders | 28 (10.0) | 15 (5.1) | 67 (3.7) | 402 (5.7) | 483 (6.3) | 338 (7.7) | 68 (8.7) |  | 1,401 (6.3) |
| **Female** | **339 (100.0)** | **683 (100.0)** | **5,425 (100.0)** | **12,461 (100.0)** | **8,653 (100.0)** | **3,946 (100.0)** | **521 (100.0)** |  | **32,028 (100.0)** |
| Mental health diagnosis | 116 (34.2) | 212 (31.0) | 1,714 (31.6) | 5,375 (43.1) | 4,142 (47.9) | 1,832 (46.4) | 221 (42.4) |  | 13,612 (42.5) |
| Organic mental disorder | 2 (0.6) | 5 (0.7) | 23 (0.4) | 99 (0.8) | 89 (1.0) | 44 (1.1) | 18 (3.5) |  | 280 (0.9) |
| Substance use disorder | 0 (0.0) | 0 (0.0) | 19 (0.4) | 56 (0.4) | 33 (0.4) | 17 (0.4) | 3 (0.6) |  | 128 (0.4) |
| Serious mental disorder | 4 (1.2) | 19 (2.8) | 88 (1.6) | 357 (2.9) | 246 (2.8) | 134 (3.4) | 18 (3.5) |  | 866 (2.7) |
| Depression | 64 (18.9) | 113 (16.5) | 951 (17.5) | 3,041 (24.4) | 2,366 (27.3) | 992 (25.1) | 117 (22.5) |  | 7,644 (23.9) |
| Anxiety | 57 (16.8) | 128 (18.7) | 1,159 (21.4) | 3,877 (31.1) | 2,967 (34.3) | 1,315 (33.3) | 142 (27.3) |  | 9,645 (30.1) |
| Other mental disorders | 36 (10.6) | 32 (4.7) | 216 (4.0) | 802 (6.4) | 636 (7.4) | 288 (7.3) | 35 (6.7) |  | 2,045 (6.4) |
| **Both sexes** | **620 (100.0)** | **976 (100.0)** | **7,252 (100.0)** | **19,523 (100.0)** | **16,362 (100.0)** | **8,340 (100.0)** | **1,305 (100.0)** |  | **54,378 (100.0)** |
| Mental health diagnosis | 189 (30.5) | 290 (29.7) | 2,185 (30.1) | 7,592 (38.9) | 6,785 (41.5) | 3,253 (39.0) | 449 (34.4) |  | 20,743 (38.1) |
| Organic mental disorder | 4 (0.6) | 7 (0.7) | 33 (0.5) | 152 (0.8) | 167 (1.0) | 93 (1.1) | 32 (2.5) |  | 488 (0.9) |
| Substance use disorder | 2 (0.3) | 6 (0.6) | 57 (0.8) | 177 (0.9) | 128 (0.8) | 55 (0.7) | 4 (0.3) |  | 429 (0.8) |
| Serious mental disorder | 4 (0.6) | 24 (2.5) | 124 (1.7) | 473 (2.4) | 376 (2.3) | 209 (2.5) | 25 (1.9) |  | 1,235 (2.3) |
| Depression | 90 (14.5) | 156 (16.0) | 1,181 (16.3) | 4,124 (21.1) | 3,645 (22.3) | 1,605 (19.2) | 199 (15.2) |  | 11,000 (20.2) |
| Anxiety | 102 (16.5) | 165 (16.9) | 1,468 (20.2) | 5,324 (27.3) | 4,702 (28.7) | 2,213 (26.5) | 274 (21.0) |  | 14,248 (26.2) |
| Other mental disorders | 64 (10.3) | 47 (4.8) | 283 (3.9) | 1,204 (6.2) | 1,119 (6.8) | 626 (7.5) | 103 (7.9) |  | 3,446 (6.3) |

**Table S2: Predicted probabilities of being in each of the four adherence groups by sex, age, and mental health diagnoses at baseline**

| **Male, % (95 confidence intervals)** | | | | | | | | | | | | | | | |
| --- | --- | --- | --- | --- | --- | --- | --- | --- | --- | --- | --- | --- | --- | --- | --- |
|  | No mental illness | | | | | | |  | Mental illness | | | | | | |
|  | 15–19 years | 20–24 years | 25–34 years | 35–44 years | 45–54 years | 55–64 years | 65+ years |  | 15–19 years | 20–24 years | 25–34 years | 35–44 years | 45–54 years | 55–64 years | 65+ years |
| Continuous non-adherence | 6.8%  (3.9–9.6) | 21.9%  (12.0–31.9) | 11.2%  (9.9–12.5) | 5.2%  (4.6–5.8) | 4.7%  (4.0–5.3) | 4.9%  (3.8–6.1) | 4.8%  (1.0–8.6) |  | 11.2%  (6.6–15.8) | 32.7%  (19.7–45.7) | 18.5%  (16.1–20.9) | 9.1%  (7.9–10.4) | 8.3%  (7.1–9.6) | 8.8%  (6.7–10.9) | 8.9%  (2.2–15.6) |
| Increasing adherence | 10.6%  (7.0–14.3) | 9.2%  (2.2–16.1) | 7.0%  (5.9–8.1) | 6.9%  (6.2–7.5) | 5.5%  (4.8–6.2) | 5.8%  (4.5–7.1) | 3.3%  (0.1–6.5) |  | 13.8%  (9.1–18.5) | 10.7%  (2.6–18.9) | 9.0%  (7.5–10.6) | 9.4%  (8.2–10.7) | 7.8%  (6.6–9.0) | 8.2%  (6.2–10.1) | 4.8%  (0.2–9.4) |
| Decreasing adherence | 22.1%  (17.2–27.0) | 18.6%  (9.1–28.2) | 17.1%  (15.5–18.7) | 14.4%  (13.5–15.4) | 11.4%  (10.5–12.4) | 12.0%  (10.2–13.8) | 5.0%  (1.1–8.9) |  | 25.5%  (19.8–31.1) | 19.4%  (9.4–29.4) | 19.7%  (17.5–21.9) | 17.6%  (16.0–19.2) | 14.3%  (12.8–15.8) | 14.9%  (12.5–17.3) | 6.5%  (1.5–11.4) |
| Continuous high adherence | 60.5%  (54.7–66.3) | 50.3%  (38.0–62.6) | 64.7%  (62.6–66.7) | 73.5%  (72.3–74.7) | 78.4%  (77.1–79.6) | 77.3%  (75.0–79.6) | 86.9%  (80.9–92.9) |  | 49.5%  (43.3–55.8) | 37.2%  (25.5–48.8) | 52.8%  (50.1–55.5) | 63.8%  (61.8–65.8) | 69.6%  (67.5–71.6) | 68.2%  (65.0–71.3) | 79.8%  (71.2–88.5) |
| **Female, % (95 confidence intervals)** | | | | | | | | | | | | | | | |
|  | No mental illness | | | | | | |  | Mental illness | | | | | | |
|  | 15–19 years | 20–24 years | 25–34 years | 35–44 years | 45–54 years | 55–64 years | 65+ years |  | 15–19 years | 20–24 years | 25–34 years | 35–44 years | 45–54 years | 55–64 years | 65+ years |
| Continuous non-adherence | 10.3%  (7.1–13.5) | 16.7%  (13.0–20.4) | 11.4%  (10.6–12.2) | 5.5%  (4.9–6.0) | 3.0%  (2.5–3.5) | 2.6%  (1.6–3.5) | 3.7%  (0.1–7.3) |  | 16.3%  (11.4–21.1) | 25.5%  (20.2–30.9) | 18.9%  (17.2–20.7) | 9.7%  (8.6–10.8) | 5.5%  (4.5–6.5) | 4.8%  (3.0–6.6) | 7.1%  (0.5–13.7) |
| Increasing adherence | 12.3%  (8.8–15.8) | 14.0%  (10.5–17.5) | 8.0%  (7.3–8.7) | 5.2%  (4.7–5.7) | 3.6%  (3.1–4.2) | 3.4%  (2.3–4.6) | 1.0%  (-0.9–2.9) |  | 15.2%  (10.9–19.6) | 16.8%  (12.5–21.1) | 10.4%  (9.1–11.6) | 7.4%  (6.4–8.3) | 5.3%  (4.4–6.2) | 5.0%  (3.3–6.7) | 1.4%  (0.0–4.2) |
| Decreasing adherence | 25.7%  (21.0–30.5) | 17.4%  (13.6–21.2) | 13.7%  (12.9–14.6) | 10.9%  (10.2–11.6) | 9.0%  (8.2–9.9) | 9.0%  (7.2–10.8) | 5.9%  (1.3–10.5) |  | 28.3%  (23.0–33.5) | 18.5%  (14.3–22.7) | 15.9%  (14.5–17.3) | 13.6%  (12.4–14.8) | 11.7%  (10.4–13.0) | 11.7%  (9.3–14.2) | 7.8%  (1.8–13.7) |
| Continuous high adherence | 51.6%  (46.2–57.0) | 51.9%  (46.8–56.9) | 66.8%  (65.6–68.0) | 78.4%  (77.4–79.3) | 84.4%  (83.3–85.5) | 85.0%  (82.7–87.3) | 89.4%  (83.5–95.4) |  | 40.2%  (34.8–45.7) | 39.2%  (34.1–44.3) | 54.8%  (52.8–56.8) | 69.3%  (67.6–71.0) | 77.5%  (75.8–79.3) | 78.5%  (75.3–81.7) | 83.7%  (75.0–92.4) |

**Table S3: Viral suppression rates by sex, age group, and mental health status at two years after baseline**

|  |  | **Viral load <400 copies/mL, % (95% CI)** | |  | **Viral load <100 copies/mL, % (95% CI)** | |  | **Viral load <1000 copies/mL, % (95% CI)** | |
| --- | --- | --- | --- | --- | --- | --- | --- | --- | --- |
| Sex | Age group, years | No mental health diagnosis | Mental health diagnosis |  | No mental health diagnosis | Mental health diagnosis |  | No mental health diagnosis | Mental health diagnosis |
| Male | 15–19 | 60.1% (50.3–69.8) | 57.7% (43.3–72.0) |  | 56.1% (47.1–65.1) | 56.3% (44.1–68.5) |  | 66.6% (57.5–75.8) | 64.6% (51.8–77.5) |
| Male | 20–24 | 65.8% (53.6–77.9) | 61.5% (44.2–78.8) |  | 58.7% (46.7–70.6) | 58.4% (42.6–74.1) |  | 67.8% (55.8–79.7) | 66.1% (49.8–82.4) |
| Male | 25–34 | 83.0% (80.4–85.7) | 80.4% (76.6–84.2) |  | 80.3% (77.7–82.8) | 78.7% (75.3–82.2) |  | 84.5% (82.0–87.0) | 81.7% (78.1–85.4) |
| Male | 35–44 | 84.6% (83.0–86.2) | 81.3% (79.0–83.7) |  | 81.1% (79.5–82.6) | 78.8% (76.8–80.8) |  | 85.7% (84.2–87.2) | 83.2% (81.2–85.2) |
| Male | 45–54 | 87.3% (85.9–88.7) | 84.7% (82.7–86.7) |  | 83.7% (82.3–85.0) | 81.1% (79.3–82.9) |  | 88.8% (87.6–90.1) | 86.8% (85.2–88.5) |
| Male | 55–64 | 88.7% (87.1–90.3) | 87.6% (85.5–89.8) |  | 84.2% (82.5–85.9) | 83.6% (81.4–85.7) |  | 90.2% (88.7–91.7) | 89.9% (88.1–91.7) |
| Male | 65+ | 90.6% (87.4–93.9) | 88.9% (84.1–93.7) |  | 87.3% (83.8–90.8) | 86.1% (81.3–90.8) |  | 92.2% (89.3–95.2) | 90.6% (86.2–94.9) |
| Female | 15–19 | 56.7% (46.0–67.4) | 53.2% (39.4–67.1) |  | 48.6% (38.7–58.5) | 46.4% (33.9–59.0) |  | 61.3% (51.3–71.3) | 58.3% (45.5–71.1) |
| Female | 20–24 | 65.0% (54.8–75.2) | 60.0% (46.7–73.2) |  | 62.6% (53.4–71.8) | 60.6% (49.4–71.8) |  | 70.7% (61.0–80.3) | 68.6% (57.1–80.1) |
| Female | 25–34 | 86.7% (85.0–88.3) | 84.4% (82.2–86.5) |  | 84.2% (82.6–85.8) | 82.2% (80.2–84.1) |  | 88.0% (86.5–89.6) | 85.7% (83.7–87.6) |
| Female | 35–44 | 89.3% (88.2–90.4) | 86.8% (85.3–88.3) |  | 87.0% (85.9–88.0) | 84.8% (83.5–86.0) |  | 90.6% (89.6–91.5) | 88.8% (87.6–89.9) |
| Female | 45–54 | 93.1% (92.2–94.0) | 91.6% (90.4–92.7) |  | 90.8% (90.0–91.7) | 88.9% (87.8–90.0) |  | 94.2% (93.5–95.0) | 93.1% (92.2–94.0) |
| Female | 55–64 | 94.7% (93.7–95.7) | 94.1% (93.0–95.3) |  | 92.1% (91.0–93.3) | 91.5% (90.2–92.7) |  | 95.5% (94.6–96.4) | 95.3% (94.3–96.2) |
| Female | 65+ | 96.0% (94.0–98.1) | 95.2% (92.6–97.8) |  | 92.9% (90.2–95.6) | 91.8% (88.5–95.1) |  | 96.9% (95.2–98.6) | 96.2% (93.8–98.5) |

Abbreviations: CI=confidence interval

**Table S4: Sensitivity analysis of associations between mental health diagnoses and non-adherence**

|  | **Primary analysis** |  | **Sensitivity analyses** | | |
| --- | --- | --- | --- | --- | --- |
|  | CMA <80%  aRR (95% CI) |  | CMA <70%  aRR (95% CI) | CMA <90%  aRR (95% CI) | CMA <80%  Repeated mental health diagnoses, aRR (95% CI) |
| Mental health diagnosis |  |  |  |  |  |
| Organic mental disorder | 1.31 (1.12–1.54) |  | 1.49 (1.24–1.78) | 1.21 (1.06–1.38) | 1.30 (0.98–1.71) |
| Substance use disorder | 1.60 (1.41–1.83) |  | 1.66 (1.41–1.94) | 1.51 (1.36–1.68) | 1.60 (1.28–1.99) |
| Serious mental disorder | 1.23 (1.11–1.36) |  | 1.26 (1.12–1.42) | 1.19 (1.09–1.29) | 1.12 (0.97–1.29) |
| Depression | 1.21 (1.17–1.25) |  | 1.23 (1.17–1.28) | 1.17 (1.14–1.21) | 1.20 (1.15–1.25) |
| Anxiety | 1.22 (1.18–1.26) |  | 1.23 (1.19–1.28) | 1.19 (1.16–1.22) | 1.19 (1.15–1.24) |
| Other mental health diagnoses | 1.10 (1.03–1.17) |  | 1.13 (1.05–1.21) | 1.07 (1.02–1.13) | 0.96 (0.86–1.07) |
| Any mental health diagnosis | 1.21 (1.18–1.25) |  | 1.23 (1.19–1.28) | 1.18 (1.15–1.20) | 1.18 (1.14–1.22) |

Risk ratios were adjusted for years since baseline, age group, and sex.

Abbreviations: CMA=cumulative medication availability, aRR=adjusted risk ratio, CI=confidence interval

**Table S5: Sensitivity analysis of associations between mental health diagnoses and viral non-suppression**

|  | **Primary analysis** |  | **Sensitivity analyses** | | |
| --- | --- | --- | --- | --- | --- |
|  | Viral load >400 copies/mL  aRR (95% CI) |  | Viral load >100 copies/mL  aRR (95% CI) | Viral load >1000 copies/mL  aRR (95% CI) | Viral load >400 copies/mL  Repeated mental health diagnoses  aRR (95% CI) |
| Mental health diagnosis |  |  |  |  |  |
| Organic mental disorder | 1.78 (1.41–2.24) |  | 1.74 (1.45–2.09) | 1.74 (1.35–2.25) | 1.90 (1.30–2.77) |
| Substance use disorder | 1.82 (1.42–2.34) |  | 1.69 (1.37–2.09) | 1.88 (1.44–2.46) | 2.06 (1.38–3.08) |
| Serious mental disorder | 1.54 (1.30–1.82) |  | 1.48 (1.29–1.71) | 1.62 (1.36–1.94) | 1.53 (1.23–1.90) |
| Depression | 1.26 (1.17–1.35) |  | 1.23 (1.16–1.30) | 1.29 (1.20–1.39) | 1.30 (1.20–1.41) |
| Anxiety | 1.11 (1.04–1.19) |  | 1.08 (1.02–1.14) | 1.10 (1.03–1.18) | 1.14 (1.05–1.23) |
| Other mental health diagnoses | 1.10 (0.98–1.23) |  | 1.07 (0.98–1.18) | 1.05 (0.93–1.19) | 1.01 (0.84–1.21) |
| Any mental health diagnosis | 1.16 (1.09–1.24) |  | 1.14 (1.08–1.20) | 1.17 (1.09–1.24) | 1.21 (1.13–1.30) |

RRs were adjusted for years since baseline, age group, and sex.

Abbreviations: aRR=adjusted risk ratio, CI=confidence interval

**Text S1: Detailed description of statistical methods to model non-adherence and viral non-suppression**

To model non-adherence, we split participant follow-up time into consecutive 12-month intervals, estimated the mean adherence during each interval, and dichotomised continuous adherence values at prespecified non-adherence thresholds, such as 80%. The analysis dataset contained one binary outcome for non-adherence for each participant and each completed year of follow-up. Using this dataset, we estimated adjusted risk ratios for factors associated with non-adherence using modified mixed-effects Poisson regression models with robust standard errors and a random intercept at patient-level. First, we fitted seven adjusted models to estimate risk ratios for each of the six groups of mental health diagnoses and any mental disorder adjusting for years since baseline, age, sex, and an interaction term between age and sex. Next, we fitted a model including an indicator for each of the six groups of mental health diagnoses, years since baseline, age, sex, and an interaction term between age and sex. We used contrasts to estimate differences in the risk of non-adherence between age groups, males and females, and participants with and without mental health diagnoses. Poisson regression models were used to overcome issues with the convergence of log-binomial models. We dichotomised adherence scores because data were heavily left-skewed.

In analysis of factors associated with viral non-suppression, the dataset contained one binary outcome for each participant and viral load test result. We fitted the models described above to estimate adjusted risk ratios for factors associated with viral non-suppression. In addition, we fitted seven models adjusting risk ratios for each group of mental health diagnoses for cumulative medication availability, age, sex, and year since baseline. We estimated differences in the risk of viral non-suppression between age groups, males and females, and participants with and without mental health diagnoses using contrasts. Finally, we estimated and plotted the probability of viral suppression (viral load <400 copies) at two years after baseline for people with and without mental health diagnoses by age and sex using predictive margins and a model including binary indicators for any mental disorder and sex, categorical variables for years since baseline, age, and interaction terms between age and sex, any mental disorder and sex, and any mental disorder and age.
